# 1,2-Bis[(2-hydr­oxy-3-methoxy­benzyl­idene)hydrazono]-1,2-diphenyl­ethane

**DOI:** 10.1107/S1600536808014049

**Published:** 2008-05-17

**Authors:** Xiu-Ying Zhang, Hui Ma, Jiu-Li Chang, Xin-Cheng Wu

**Affiliations:** a Henan Normal University, College of Chemistry and Environmental Science, Xinxiang 453002, People’s Republic of China

## Abstract

The title compound, C_30_H_26_N_4_O_4_, was synthesized by the reaction of benzyl dihydrazone and 2-hydr­oxy-3-methoxy­benzaldehyde in ethanol. In the crystal strucutre, the mol­ecule is centrosymmetric. The structure displays two symmetry-related intra­molecular O—H⋯N hydrogen bonds.

## Related literature

For related literature, see: Pankaj *et al.* (2000 ▶); Senjuti *et al.* (2006 ▶); Shubhamoy *et al.* (2003 ▶); Boudalis *et al.* (2004 ▶); Veauthier *et al.* (2004 ▶).
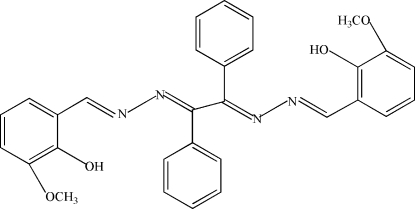


## Experimental

### 

#### Crystal data

C_30_H_26_N_4_O_4_
*M* *_r_* = 506.55
Monoclinic, 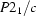

*a* = 8.3732 (11) Å
*b* = 12.7267 (16) Å
*c* = 12.4229 (16) Å
β = 98.188 (2)°
*V* = 1310.3 (3) Å^3^
*Z* = 2
Mo *K*α radiation
μ = 0.09 mm^−1^
*T* = 291 (2) K
0.36 × 0.19 × 0.11 mm

#### Data collection

Bruker SMART CCD area-detector diffractometer
Absorption correction: multi-scan (*SADABS*; Sheldrick, 1996 ▶) *T*_min_ = 0.969, *T*_max_ = 0.991
9588 measured reflections
2437 independent reflections
1543 reflections with *I* > 2σ(*I*)
*R*_int_ = 0.030

#### Refinement

*R*[*F* ^2^ > 2σ(*F* ^2^)] = 0.041
*wR*(*F* ^2^) = 0.117
*S* = 1.01
2437 reflections
174 parameters
H-atom parameters constrained
Δρ_max_ = 0.11 e Å^−3^
Δρ_min_ = −0.15 e Å^−3^

### 

Data collection: *SMART* (Bruker, 2001 ▶); cell refinement: *SAINT* (Bruker, 2001 ▶); data reduction: *SAINT*; program(s) used to solve structure: *SHELXS97* (Sheldrick, 2008 ▶); program(s) used to refine structure: *SHELXL97* (Sheldrick, 2008 ▶); molecular graphics: *SHELXTL* (Sheldrick, 2008 ▶); software used to prepare material for publication: *SHELXTL* .

## Supplementary Material

Crystal structure: contains datablocks I, global. DOI: 10.1107/S1600536808014049/kp2163sup1.cif

Structure factors: contains datablocks I. DOI: 10.1107/S1600536808014049/kp2163Isup2.hkl

Additional supplementary materials:  crystallographic information; 3D view; checkCIF report

## Figures and Tables

**Table 1:** Hydrogen-bond geometry (Å, °)

*D*—H⋯*A*	*D*—H	H⋯*A*	*D*⋯*A*	*D*—H⋯*A*
O2—H2⋯N1	0.82	1.91	2.6350 (18)	146

**Fig. 1.**
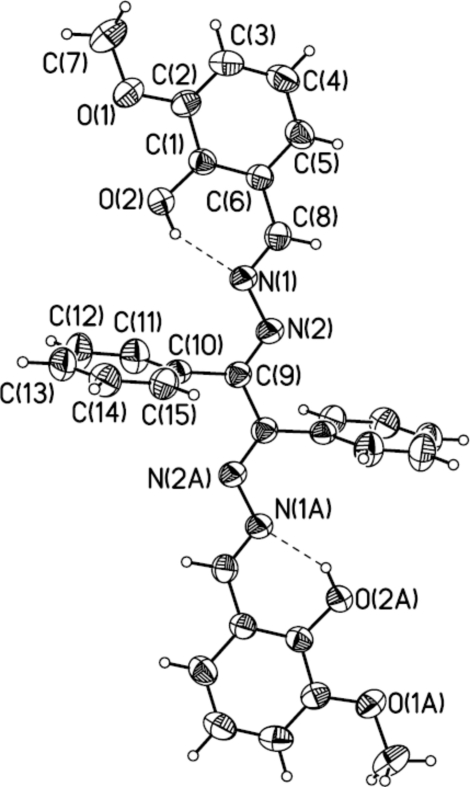
The molecular structure of (I) with atom labels and the 30% probability displacement ellipsoids for non-H atoms. The symmetry related atoms (A) are generated by symmetry operation: 1 - x, -y, 2 - z.

## supplementary crystallographic information

### Comment

The design of multidentate Schiff-base ligands and their metal complexes are of great interest in the last few years (Boudalis *et al.*, 2004; Veauthier *et al.*, 2224; Pal *et al.*, 2000). The crystal structure determination of the title compound, (I), has been carried out in order to elucidate its molecular conformation. The molecule of the compound, (I), (Fig.1) is centrosymmetric with a centre of inversion in the middle of C9—C9 A bond. The two benzene rings (C10→C15 and C10 A→C15 A) are parallel. The dihedral angle between the benzene ring (C10→C15) and the least-squares best plane (C1→C6, C8, N1, O1, O2, r.m.s.= 0.0262 Å) is 74.2°. The bond lengths of C9—C9 A, N2—C9, N2—N1, N1—C8 are 1.474 (3) Å, 1.289 (2) Å, 1.4013 (19) Å, and 1.284 (2) Å, respectively. All the angles and bond lengths are within normal range (Pankaj *et al.*, 2000). The symmetry related intramolecular hydrogen bonds O—H···N are observed (Fig. 1, Table 1).

### Experimental

All reagents were of AR grade, available commercially and used without further purification. The mixture of benzyl dihydrazone (0.595 g, 2.5 mmol), 2-hydroxy-3-methoxybenzaldehyde (0.76 g, 5 mmol) was heated and refluxed in ethanol (20 ml for 3 h, and then the resulting solution was cooled to room temperature. After filtration, the filtrate was allowed to stand at room temperature. Upon slow evaporation, yellow block crystals suitable for X-ray diffraction analysis were isolated after three days.

### Refinement

The H atoms were positioned geometrically and refined using the riding-model approximation, with C—H = 0.93 or 0.96 Å and O—H = 0.82 Å and *U*_iso_(H) = 1.2Ueq(carrier) or *U*_iso_(H) = 1.5Ueq(methyl carrier).

### Crystal data

**Table:**

C_30_H_26_N_4_O_4_	*F*_000_ = 532
*M**_r_* = 506.55	*D*_x_ = 1.284 Mg m^−^^3^
Monoclinic, *P*2_1_/*c*	Mo *K*α radiation λ = 0.71073 Å
*a* = 8.3732 (11) Å	Cell parameters from 1614 reflections
*b* = 12.7267 (16) Å	θ = 2.5–22.5º
*c* = 12.4229 (16) Å	µ = 0.09 mm^−^^1^
β = 98.188 (2)º	*T* = 291 (2) K
*V* = 1310.3 (3) Å^3^	Block, yellow
*Z* = 2	0.36 × 0.19 × 0.11 mm

### Data collection

**Table:**

Bruker SMART CCD area-detector diffractometer	2437 independent reflections
Radiation source: fine-focus sealed tube	1543 reflections with *I* > 2σ(*I*)
Monochromator: graphite	*R*_int_ = 0.030
*T* = 291(2) K	θ_max_ = 25.5º
φ and ω scans	θ_min_ = 2.5º
Absorption correction: multi-scan(SADABS; Sheldrick, 1996)	*h* = −10→10
*T*_min_ = 0.969, *T*_max_ = 0.991	*k* = −15→15
9588 measured reflections	*l* = −15→15

### Refinement

**Table:**

Refinement on *F*^2^	Secondary atom site location: difference Fourier map
Least-squares matrix: full	Hydrogen site location: inferred from neighbouring sites
*R*[*F*^2^ > 2σ(*F*^2^)] = 0.041	H-atom parameters constrained
*wR*(*F*^2^) = 0.117	*w* = 1/[σ^2^(*F*_o_^2^) + (0.0507*P*)^2^ + 0.1624*P*] where *P* = (*F*_o_^2^ + 2*F*_c_^2^)/3
*S* = 1.02	(Δ/σ)_max_ < 0.001
2437 reflections	Δρ_max_ = 0.11 e Å^−^^3^
174 parameters	Δρ_min_ = −0.15 e Å^−^^3^
Primary atom site location: structure-invariant direct methods	Extinction correction: none

### Special details

**Table:**

Geometry. All e.s.d.'s (except the e.s.d. in the dihedral angle between two l.s. planes)are estimated using the full covariance matrix. The cell e.s.d.'s are takeninto account individually in the estimation of e.s.d.'s in distances, anglesand torsion angles; correlations between e.s.d.'s in cell parameters are onlyused when they are defined by crystal symmetry. An approximate (isotropic)treatment of cell e.s.d.'s is used for estimating e.s.d.'s involving l.s. planes.
Refinement. Refinement of *F*^2^ against ALL reflections. The weighted *R*-factor *wR* andgoodness of fit *S* are based on *F*^2^, conventional *R*-factors *R* are basedon *F*, with *F* set to zero for negative *F*^2^. The threshold expression of*F*^2^ > σ(*F*^2^) is used only for calculating *R*-factors(gt) *etc*. and isnot relevant to the choice of reflections for refinement. *R*-factors basedon *F*^2^ are statistically about twice as large as those based on *F*, and *R*-factors based on ALL data will be even larger.

### Fractional atomic coordinates and isotropic or equivalent isotropic displacement parameters (Å^2^)

**Table:**

	*x*	*y*	*z*	*U*_iso_*/*U*_eq_	
O1	0.47861 (17)	0.23761 (11)	0.56864 (11)	0.0720 (4)	
O2	0.30818 (16)	0.17749 (10)	0.71864 (11)	0.0648 (4)	
H2	0.2592	0.1502	0.7640	0.097*	
N1	0.24208 (19)	0.03065 (11)	0.85518 (12)	0.0567 (4)	
N2	0.15856 (19)	−0.02470 (11)	0.92719 (12)	0.0578 (4)	
C1	0.4277 (2)	0.11223 (14)	0.69686 (15)	0.0503 (4)	
C2	0.5195 (2)	0.14299 (15)	0.61656 (15)	0.0549 (5)	
C3	0.6395 (2)	0.07799 (17)	0.59099 (17)	0.0661 (6)	
H3	0.6997	0.0977	0.5369	0.079*	
C4	0.6719 (2)	−0.01642 (16)	0.64473 (18)	0.0708 (6)	
H4	0.7542	−0.0592	0.6268	0.085*	
C5	0.5847 (2)	−0.04748 (15)	0.72363 (17)	0.0632 (5)	
H5	0.6082	−0.1108	0.7597	0.076*	
C6	0.4592 (2)	0.01623 (13)	0.75048 (14)	0.0497 (4)	
C7	0.5529 (3)	0.26528 (19)	0.47601 (16)	0.0854 (7)	
H7A	0.6672	0.2722	0.4972	0.128*	
H7B	0.5094	0.3308	0.4468	0.128*	
H7C	0.5320	0.2114	0.4217	0.128*	
C8	0.3612 (2)	−0.02192 (14)	0.82838 (15)	0.0551 (5)	
H8	0.3851	−0.0871	0.8605	0.066*	
C9	0.0459 (2)	0.02843 (13)	0.96279 (14)	0.0499 (4)	
C10	0.0056 (2)	0.14018 (13)	0.93434 (15)	0.0494 (5)	
C11	−0.0776 (3)	0.16613 (16)	0.83395 (17)	0.0721 (6)	
H11	−0.1114	0.1133	0.7842	0.087*	
C12	−0.1110 (3)	0.26938 (19)	0.8066 (2)	0.0839 (7)	
H12	−0.1656	0.2859	0.7381	0.101*	
C13	−0.0644 (3)	0.34772 (17)	0.8795 (2)	0.0753 (6)	
H13	−0.0891	0.4174	0.8616	0.090*	
C14	0.0186 (3)	0.32303 (16)	0.97909 (19)	0.0697 (6)	
H14	0.0514	0.3763	1.0286	0.084*	
C15	0.0545 (2)	0.21959 (15)	1.00694 (16)	0.0600 (5)	
H15	0.1117	0.2036	1.0747	0.072*	

### Atomic displacement parameters (Å^2^)

**Table:**

	*U*^11^	*U*^22^	*U*^33^	*U*^12^	*U*^13^	*U*^23^
O1	0.0830 (10)	0.0687 (9)	0.0680 (9)	−0.0023 (7)	0.0232 (8)	0.0159 (7)
O2	0.0686 (9)	0.0565 (8)	0.0741 (10)	0.0137 (7)	0.0271 (7)	0.0126 (7)
N1	0.0599 (10)	0.0488 (9)	0.0657 (10)	0.0003 (8)	0.0242 (8)	0.0066 (8)
N2	0.0613 (10)	0.0504 (9)	0.0656 (10)	−0.0009 (8)	0.0230 (8)	0.0070 (8)
C1	0.0488 (10)	0.0489 (10)	0.0540 (11)	−0.0016 (8)	0.0104 (8)	−0.0042 (9)
C2	0.0572 (12)	0.0540 (11)	0.0540 (11)	−0.0104 (9)	0.0100 (9)	−0.0018 (9)
C3	0.0598 (13)	0.0732 (15)	0.0701 (14)	−0.0096 (11)	0.0254 (11)	−0.0097 (11)
C4	0.0599 (13)	0.0699 (15)	0.0872 (15)	0.0049 (11)	0.0260 (12)	−0.0096 (12)
C5	0.0603 (13)	0.0521 (11)	0.0791 (14)	0.0048 (9)	0.0164 (11)	−0.0057 (10)
C6	0.0500 (11)	0.0430 (10)	0.0576 (11)	−0.0031 (8)	0.0129 (9)	−0.0038 (8)
C7	0.115 (2)	0.0852 (16)	0.0582 (13)	−0.0224 (14)	0.0215 (13)	0.0074 (11)
C8	0.0617 (12)	0.0413 (10)	0.0632 (12)	−0.0007 (9)	0.0122 (10)	0.0006 (8)
C9	0.0516 (11)	0.0463 (10)	0.0527 (11)	−0.0035 (8)	0.0108 (9)	0.0014 (8)
C10	0.0461 (10)	0.0485 (10)	0.0561 (11)	−0.0019 (8)	0.0158 (9)	0.0023 (9)
C11	0.0846 (16)	0.0638 (14)	0.0646 (14)	0.0045 (11)	−0.0004 (12)	−0.0023 (11)
C12	0.0991 (19)	0.0722 (15)	0.0766 (15)	0.0168 (13)	−0.0001 (13)	0.0155 (13)
C13	0.0737 (15)	0.0544 (13)	0.0999 (18)	0.0071 (11)	0.0203 (14)	0.0188 (13)
C14	0.0718 (14)	0.0490 (12)	0.0898 (16)	−0.0081 (10)	0.0162 (12)	−0.0057 (11)
C15	0.0643 (13)	0.0524 (12)	0.0626 (12)	−0.0043 (10)	0.0063 (10)	0.0004 (10)

### Geometric parameters (Å, °)

**Table:**

O1—C2	1.365 (2)	C7—H7A	0.9600
O1—C7	1.428 (2)	C7—H7B	0.9600
O2—C1	1.357 (2)	C7—H7C	0.9600
O2—H2	0.8200	C8—H8	0.9300
N1—C8	1.284 (2)	C9—C9^i^	1.474 (3)
N1—N2	1.4013 (19)	C9—C10	1.492 (2)
N2—C9	1.289 (2)	C10—C15	1.377 (3)
C1—C6	1.399 (2)	C10—C11	1.379 (3)
C1—C2	1.399 (2)	C11—C12	1.376 (3)
C2—C3	1.374 (3)	C11—H11	0.9300
C3—C4	1.382 (3)	C12—C13	1.366 (3)
C3—H3	0.9300	C12—H12	0.9300
C4—C5	1.362 (3)	C13—C14	1.366 (3)
C4—H4	0.9300	C13—H13	0.9300
C5—C6	1.404 (2)	C14—C15	1.383 (3)
C5—H5	0.9300	C14—H14	0.9300
C6—C8	1.439 (2)	C15—H15	0.9300
			
C2—O1—C7	117.26 (16)	H7A—C7—H7C	109.5
C1—O2—H2	109.5	H7B—C7—H7C	109.5
C8—N1—N2	112.36 (15)	N1—C8—C6	122.49 (17)
C9—N2—N1	114.29 (15)	N1—C8—H8	118.8
O2—C1—C6	122.30 (16)	C6—C8—H8	118.8
O2—C1—C2	117.81 (16)	N2—C9—C9^i^	115.5 (2)
C6—C1—C2	119.88 (16)	N2—C9—C10	124.79 (15)
O1—C2—C3	125.21 (17)	C9^i^—C9—C10	119.7 (2)
O1—C2—C1	115.38 (16)	C15—C10—C11	118.76 (17)
C3—C2—C1	119.40 (18)	C15—C10—C9	120.53 (17)
C2—C3—C4	120.76 (19)	C11—C10—C9	120.69 (17)
C2—C3—H3	119.6	C12—C11—C10	120.7 (2)
C4—C3—H3	119.6	C12—C11—H11	119.6
C5—C4—C3	120.76 (19)	C10—C11—H11	119.6
C5—C4—H4	119.6	C13—C12—C11	120.3 (2)
C3—C4—H4	119.6	C13—C12—H12	119.9
C4—C5—C6	119.96 (19)	C11—C12—H12	119.9
C4—C5—H5	120.0	C14—C13—C12	119.5 (2)
C6—C5—H5	120.0	C14—C13—H13	120.2
C1—C6—C5	119.22 (16)	C12—C13—H13	120.2
C1—C6—C8	121.85 (16)	C13—C14—C15	120.6 (2)
C5—C6—C8	118.84 (17)	C13—C14—H14	119.7
O1—C7—H7A	109.5	C15—C14—H14	119.7
O1—C7—H7B	109.5	C10—C15—C14	120.05 (19)
H7A—C7—H7B	109.5	C10—C15—H15	120.0
O1—C7—H7C	109.5	C14—C15—H15	120.0
			
C8—N1—N2—C9	174.82 (16)	N2—N1—C8—C6	176.37 (15)
C7—O1—C2—C3	8.0 (3)	C1—C6—C8—N1	−1.7 (3)
C7—O1—C2—C1	−171.27 (17)	C5—C6—C8—N1	−178.34 (17)
O2—C1—C2—O1	0.5 (2)	N1—N2—C9—C9^i^	178.95 (17)
C6—C1—C2—O1	179.61 (16)	N1—N2—C9—C10	−1.4 (3)
O2—C1—C2—C3	−178.83 (16)	N2—C9—C10—C15	−102.7 (2)
C6—C1—C2—C3	0.3 (3)	C9^i^—C9—C10—C15	76.9 (3)
O1—C2—C3—C4	179.79 (18)	N2—C9—C10—C11	75.6 (3)
C1—C2—C3—C4	−0.9 (3)	C9^i^—C9—C10—C11	−104.8 (2)
C2—C3—C4—C5	0.5 (3)	C15—C10—C11—C12	0.1 (3)
C3—C4—C5—C6	0.6 (3)	C9—C10—C11—C12	−178.26 (19)
O2—C1—C6—C5	179.88 (17)	C10—C11—C12—C13	−1.1 (4)
C2—C1—C6—C5	0.8 (3)	C11—C12—C13—C14	1.4 (4)
O2—C1—C6—C8	3.3 (3)	C12—C13—C14—C15	−0.6 (3)
C2—C1—C6—C8	−175.80 (16)	C11—C10—C15—C14	0.7 (3)
C4—C5—C6—C1	−1.3 (3)	C9—C10—C15—C14	179.01 (17)
C4—C5—C6—C8	175.46 (18)	C13—C14—C15—C10	−0.4 (3)

Symmetry codes: (i) −*x*, −*y*, −*z*+2.

### Hydrogen-bond geometry (Å, °)

**Table:**

*D*—H···*A*	*D*—H	H···*A*	*D*···*A*	*D*—H···*A*
O2—H2···N1	0.82	1.91	2.6350 (18)	146
